# Disparities in cancer care among sexual and gender minority adolescent and young adult patients: A scoping review

**DOI:** 10.1002/cam4.6090

**Published:** 2023-05-28

**Authors:** Christabel K. Cheung, Haelim Lee, Nina Jackson Levin, Eunju Choi, Valentina A. Ross, Yimin Geng, Bria N. Thomas, Michael E. Roth

**Affiliations:** ^1^ University of Maryland School of Social Work Baltimore Maryland USA; ^2^ University of Michigan School of Social Work and Department of Anthropology Michigan Ann Arbor USA; ^3^ Department of Nursing and MD Anderson Cancer Center University of Texas Houston Texas USA; ^4^ Research Medical Library University of Texas MD Anderson Cancer Center Houston Texas USA; ^5^ Geisinger Commonwealth School of Medicine Scranton Pennsylvania USA; ^6^ University of Texas MD Anderson Cancer Center Houston Texas USA

**Keywords:** adolescent and young adult, cancer survivors, disparities, LGBTQ+, sexual and gender minority

## Abstract

**Background:**

Adolescent and young adult cancer patients (AYAs) who are sexual and gender minorities (SGM) are a rapidly increasing population that experiences unmet cancer‐related needs. Despite emerging awareness, little is known about cancer care and outcomes for this vulnerable population. The purpose of this scoping review was to explore current knowledge and gaps in the literature on cancer care and outcomes for AYAs who identify as SGM.

**Methods:**

We reviewed empirical knowledge on SGM AYAs by identifying, describing, and critically appraising the literature to date. We conducted a comprehensive search on OVID MEDLINE, PsycINFO, and CINAHL in February 2022. Additionally, we developed and piloted a conceptual framework for appraising SGM AYA research.

**Results:**

A total of 37 articles were included in the final review. Most studies focused exclusively on SGM‐related outcomes as the primary aim of the study (81.1%, *n* = 30), whereas others included some focus on SGM‐related outcomes (18.9%, *n* = 7). The majority of studies included AYAs as part of a broader age range (86.0%, *n* = 32), and only a few studies examined exclusively AYA samples (14.0%, *n* = 5). Gaps in scientific evidence on SGM AYAs were seen across the cancer care continuum.

**Conclusion:**

Numerous gaps in knowledge of cancer care and outcomes exist for SGM AYAs diagnosed with cancer. Future efforts should fill this void with high‐quality empirical studies that reveal unknown disparities in care and outcomes and are inclusive of the intersectionality of SGM AYAs with other minoritized experiences, thereby advancing health equity in meaningful ways.

## Graphical abstract

The purpose of this scoping review was to explore current knowledge and gaps in the literature on cancer care and outcomes for adolescent and young adult cancer patients (AYA) who identify as sexual and gender minorities (SGM). We conducted a comprehensive search on OVID MEDLINE, PsycINFO, and CINAHL and developed and piloted a conceptual framework for appraising SGM AYA research. A total of 37 articles were included in the final review. Future efforts should fill existing gaps by investigating potential disparities in care and outcomes for this vulnerable population.

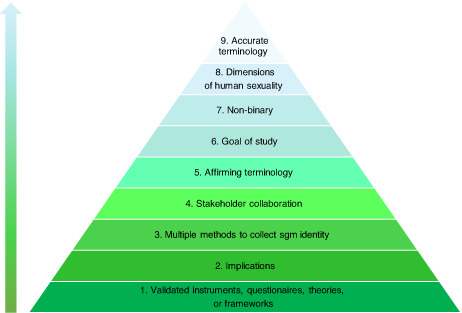

## 1 BACKGROUND

In 2022, there were an estimated 87,500 new cases of cancer diagnosed in adolescents and young adults (AYAs) between ages 15 and 39 years, comprising 4.5% of all new cancer cases in the United States (US).^1^ AYAs experience distinct disparities and challenges including gaps in diagnosis,^2^ barriers in accessing care,^2^ and potentially added complexities in the impact of cancer on career trajectories and future health.^3^ AYAs who are sexual and gender minorities (SGM)—that is, identify as lesbian, gay, bisexual, transgender, queer, and/or any other noncisgender or nonheterosexual identities (LGBTQ+)^4^—are a rapidly increasing population that experiences unmet cancer‐related needs. Nationally representative data on the distribution of SGM across age cohorts in the US are limited. In 2022, Gallup—a private analytic firm—estimated that 20.8% of Generation Z (born 1997–2003) and 10.5% of Millenials (born 1981–1996) openly identify as LGBT, whereas only 7.6% of adults born before 1980 openly identify as LGBT.^5^ Prior studies have reported that SGM cancer patients face multiple healthcare and psychosocial challenges compared with their non‐SGM counterparts^6^, ^7^, ^8^—for example, higher levels of psychological distress^7^, ^8^ and additional barriers to receiving cancer care.^6^

Despite emerging awareness of SGM AYAs as a psychosocially and medically vulnerable population and recognition of the need to improve their care,^6^, ^9^ the quality of studies addressing SGM needs and experiences can be difficult to evaluate due to incomplete reporting of key elements. Furthermore, quality assessment of individual studies plays a crucial role in building a generalizable, empirical body of literature that enables healthcare professionals to critically evaluate and apply findings in the provision of care.^10^ A conceptual framework is missing and needed to prioritize SGM AYA domains that are relevant to the assessment of research quality.

The goal of the current scoping review was to explore current knowledge and gaps in the literature on care and outcomes for AYAs who identify as SGM. Additionally, we responded to a lack of existing tools that adequately appraise the quality of the SGM literature by developing and piloting a novel conceptual framework for assessing the quality of SGM AYA research. Thus, the present review innovatively generates and defines standards for reporting SGM AYA research in the conduct of this scoping review.

## 2 METHODS

### 2.1 Terminology

Terminology used by authors in this manuscript reflects both the language found in our review of extant literature and our commitment to utilizing language that represents the current cohort of AYAs whose gender identities and sexual orientations are increasingly expansive. More specifically, we used the umbrella terms “sexual and gender minority” (SGM), “sexual orientation and gender identity” (SOGI), “lesbian, gay, bisexual, transgender, queer, plus” (LGBTQ+), and “queer” as revealed in the conduct of our review. Concurrently, we were as specific as possible in the identification of subgroups where applicable. The Human Rights Campaign's glossary of terms^11^ was our primary reference for present‐day inclusive language. The terms LGBTQ+ and SGM are used interchangeably as are the terms patient and survivor.

### 2.2 Information sources

Three members of the research team (C.K.C., H.L., and V.A.R.) initially generated search terms (Table 1), which were subsequently reviewed and vetted by all authors. The research librarian (Y.G.) conducted a comprehensive search on OVID MEDLINE, PsycINFO, and CINAHL in February 2022. Search terms used to generate results were arrived at by using keywords and synonymous index terms within our research question and by following guidance from extant reviews of health‐related topics for SGM populations.

**TABLE 1:** Search terms in scoping review of cancer care and outcomes for sexual and gender minority adolescent and young adults (AYAs).

	Search terms
1	Young Adult OR Adolescent OR adolescen* OR teen OR teenager* OR youth OR youths OR young adult* OR emerging adult*” OR young women OR young men OR AYA OR AYAs OR childhood OR young adj5 (adult* OR girl* OR boy* OR women OR men OR female OR male OR patient* OR survivor) OR pediatric OR paediatric OR Pediatrics OR ((15‐39 OR 15‐16 OR 15‐17 OR 15‐18 OR 15‐25 OR 18‐25 OR 18‐39 OR 19‐24 OR 19‐25 OR 19‐26 OR 19‐39 OR 20‐39 OR 21‐39 OR 25‐39 OR 26‐39) adj3 (year* OR age*)) [AYA population]
2	Neoplasms OR (cancer* OR carcinom* OR tumor* OR tumour* OR neoplas* OR malignan* OR metasta* OR myeloma* OR leukiemia* OR lymphoma* OR sarcoma* OR melanoma*) OR (Cancer Survivors) [Cancer]
3	1 AND 2
4	Sexual and Gender Minorities OR Bisexuality OR Homosexuality OR Transsexualism OR Gender Identity OR (GLBT* OR LBGT* OR LGBT*) OR ((sexual OR gender) adj3 (dissident* OR minorit*)) OR (sexual orientation OR sexual reassignment) OR (lesbian* OR lesbigay OR gay OR gays OR bisexual* OR asexual* OR pansexual* OR demisexual* OR androsexual* OR gynosexual* OR homosexual* OR “non‐heterosexual*” OR transgender OR transsexual* OR queer OR queers OR intersex OR gender non‐conforming OR gender affirming OR gender confirmation) OR (gender fluid OR sexually fluid OR agender OR genderless OR genderqueer* OR two‐spirit) OR (gender expansive OR gender dysphoria) OR (women who have sex with women OR men who have sex with men) OR (women who love women OR men who love men) OR Sex Reassignment Procedures OR (transmasculine OR trans masculine OR transfeminine OR trans feminine OR nonbinary OR non‐binary) [sexual gender minorities]
5	3 AND 4

### 2.3 Inclusion and exclusion criteria

Studies were included if they met the following seven criteria: (1) English language publication; (2) empirical study, that is, scientific research based on observation or experimentation; (3) reported on a defined patient sample or subsample of patients diagnosed with cancer between ages 15 and 39 years, who may be older at the time of the study; (4) human research; (5) study sample included those investigating research outcomes (i.e., endpoints) related to sexual and gender minority status; (6) examined factor(s) related to a biological, medical, and/or psychosocial aspect related to a cancer diagnosis; (7) examined patients on‐ or posttreatment. Studies with endpoints that focused on healthcare providers were excluded as were nonempirical publications such as meeting abstracts, editorials, or case reports.

### 2.4 Search procedures

Search results were compiled in Covidence (Covidence). Five reviewers (C.K.C., E.C., H.L., M.E.R., and V.A.R) participated in the title/abstract screening. Each title/abstract was screened by three reviewers, who resolved disagreements through discussion among them until a consensus was reached. If consensus could still not be reached, then all five reviewers were consulted to arrive at an agreement. A similar process was used to screen the full text of articles reviewed by a total of six reviewers (C.K.C., E.C., H.L., M.E.R., N.J.L., & V.A.R).

### 2.5 Data analysis

The authors developed a data extraction form in Microsoft Excel (Microsoft) that was populated with search results from Covidence and captured the following information for each study: primary author, year of publication, country of origin, title, study aim, study design, study start date, study end date, method(s) of recruitment, participant characteristics (sample size, age, cancer type, cancer stage, cancer phase, comparison group), study results (cancer outcomes, LGBTQ+ related outcomes, psychosocial outcomes, and physical outcomes), and reviewers' notes on key findings.

### 2.6 Quality of the evidence

**FIGURE 1:**
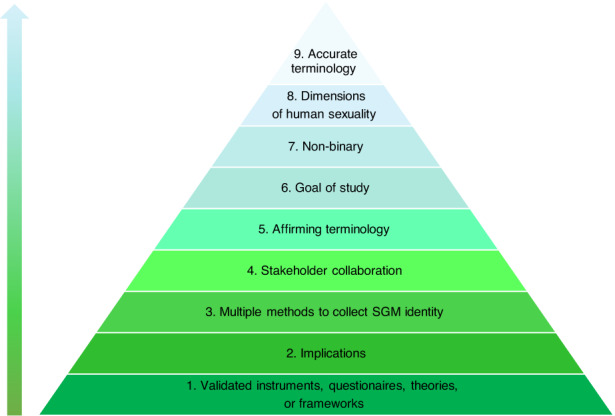
Conceptual framework of foundational progress in sexual and gender minority (SGM) and adolescent and young adult (AYA) research derived from Levin et al.'s^14^ schema for assessing sex, gender identity, and sexual orientation in oncofertility research by presenting nine essential domains for high‐quality research studies that capture SGM AYAs.

The quality of publications was first calculated by using the relevant Joanna Briggs Institute (JBI) critical appraisal tool^12^, ^13^ for the respective study design. The authors then examined SGM AYA research quality by developing and piloting a new conceptual framework that was adapted from considerations for assessing SGM oncofertility praxis^14^ (Figure 1).

## 3 RESULTS

**FIGURE 2:**
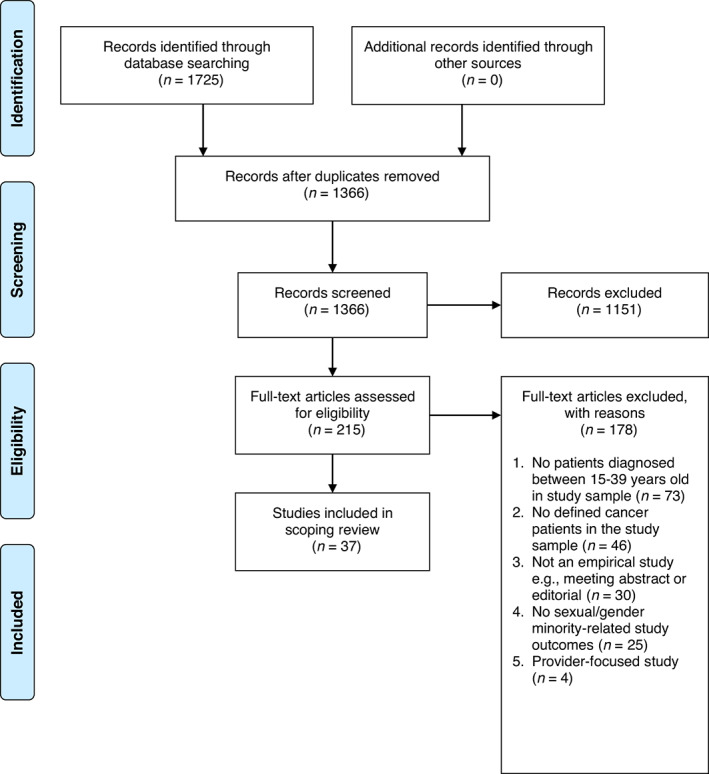
PRISMA diagram of search results and excluded articles in our scoping review of the literature on sexual and gender minority adolescent and young adult cancer care and outcomes. Adapted from Moher et al.^15^

In total, 1725 articles were retrieved. After duplicates within and between databases were removed, 1366 records remained (Figure 2). Of the 1366 articles, 1151 were excluded because they did not meet study inclusion criteria during the title and abstract review. Thereafter, 215 articles were deemed potentially relevant and underwent full‐text review. Upon completion of full‐text screening 178 articles were removed. Reasons for exclusion of articles were: no patients diagnosed between 15 and 39 years old in the study sample (*n* = 73); no defined cancer patients in the study sample (*n* = 46); not an empirical study (*n* = 30); no sexual and gender minority‐related study outcomes (*n* = 25); provider‐focused study (*n* = 4). A total of 37 articles were included in the final review (Figure 2).

### 3.1 Study characteristics

Table 1 characterizes our resulting studies (*n* = 37). The preponderance of studies included in this scoping review focused exclusively on SGM‐related outcomes as the primary aim of the study (81.1%, *n* = 30), whereas other studies included some focus on SGM‐related outcomes (18.9%, *n* = 7). Most studies included AYAs as part of a broader age range (86.5%, *n* = 32), and only a few studies used an exclusively AYA sample (13.5%, *n* = 5). Of these 5 articles with exclusively AYA samples, only one study focused exclusively on SGM AYAs—a narrative inquiry with two participants.

Methods of recruitment varied with most studies (*n* = 24) employing more than one method. Approximately one‐third of the studies used clinic visits (*n* = 11) and/or social media (*n* = 10). Other recruitment approaches included phone (*n* = 6), postal service (*n* = 5), email (*n* = 7), and other in‐person contact (*n* = 5). Considering that nearly half of studies included in our review were qualitative studies (48.6%, *n* = 18), the majority (40.5%, *n* = 15) recruited sample sizes of less than 32 participants (Table 1).

More than half of studies included LGBTQ+ participants as part of a larger sample of cancer patients (56.8%, *n* = 21), while the remaining studies examined exclusively LGBTQ+ cancer patients (43.2%, *n* = 16). Approximately one third of studies solely focused on one cancer type (breast, gynecological, colorectal, testicular, and acquired immunodeficiency syndrome [AIDS]‐defining cancer), while the majority studies represented a variety of cancer types. Of 37 studies, (48.6%, *n* = 18) were qualitative studies and (43.2%, *n* = 16) were quantitative studies. Three studies (8.1%) utilized mixed‐methods. All quantitative studies applied cross‐sectional designs (Table 2).

**TABLE 2:** Characteristics of included studies (*n* = 37).

Country	No. of articles
USA	25
UK	1
Canada	7
Australia	2
The Netherlands	1
Sweden	1
Study design	
Quantitative: cross‐sectional	16
Qualitative	18
Mixed methods	3
Sample	
˂32	15
33–100	8
101–500	5
501–1000	2
>1000	7
Exclusively lesbian, gay, bisexual, transgender, queer (LGBTQ+) participants	16
LGBTQ+ participants within a broader sample	21
Exclusively adolescent and young adult (AYA) participants	5
AYA participants within a broader sample	32
Exclusively LGBTQ+ AYAs	1
Age^a^	
Teens	13
20s	24
30s	29
Cancer type(s)^a^	
N/A	2
Leukemia	4
Hodgkin's lymphoma	4
Non‐Hodgkin's lymphoma	2
Sarcoma (e.g., osteosarcoma, Ewing's, etc.)	2
Germ cell tumors (e.g., testis, ovarian, etc.)	5
Gastric cancer (e.g., colon, stomach, etc.)	8
Multiple cancer types	11
Other cancer type(s)	24

^a^ Some studies represent more than one option within this category.

### 3.2 Biomedical outcomes

No studies reported on cancer outcomes such as overall survival, disease‐free survival, or progression‐free survival (Table 3).

**TABLE 3:** Summary of articles (*n* = 37).

Reference	Location	Study sample	Design	Primary outcomes	Sexual and gender minority (SGM) quality assessment
Alpert et al.^65^	USA	*N* = 12; patients (*n* = 7), physicians (*n* = 5); transgender women (*n* = 2), transgender man (*n* = 1), nonbinary and/or queer (*n* = 4)	Qualitative; two group interviews	Psychosocial outcomes: paternalism (from enforced gender expectations): gendered form in the clinics, no proper rooms, gowns. Stigmatization (from enforced gender expectations); denied admission to emergency psychiatric care because the patient is transgender. Self‐advocacy and allyship (resisting expectations)	Captures 2, 3, 5, 7–9
Avutu et al.^66^	USA	*N* = 24; lesbian, gay, bisexual, transgender, queer (LGBTQ+) (*n* = 5), non‐LGBTQ+ (*n* = 19)	Focus groups	Psychosocial outcomes: navigating both sexuality and gender identity during cancer treatment can be overwhelming	Captures 2, 5, 6, 8, 9
Boehmer et al.^16^	USA	*N* = 480; sexual minority (*n* = 127), heterosexual (*n* = 353)	Cross‐sectional	Psychosocial outcomes: sexual minority and heterosexual survivors rated each aspect of interpersonal care similarly. However, sexual minorities rated the overall quality of cancer care more favorably than heterosexuals. Sexual minority survivors' greater likelihood of rating care as excellent compared with heterosexual survivors might be due to differences in expectations. It is possible that sexual minority survivors in this study had low expectations of care before cancer, then perceived cancer care as better than expected; the geographic areas from which participants were recruited were denser than the SGM population	Captures 5, 6, 9
Boehmer et al.^17^	USA	*N* = 70,524; sexual minority men (*n* = 782), heterosexual men (*n* = 24,422); sexual minority women (*n* = 1149), heterosexual women (*n* = 44,171)	Cross‐sectional	Psychosocial outcomes: SGM women and SGM men with worse access to care have poorer quality of life	Captures 2–9
Boehmer et al.^18^	USA	*N* = 954,908; transgender women (*n* = 1877); transgender men (*n* = 1344); gender nonconforming (*n* = 876); cisgender men (*n* = 410,422); cisgender women (*n* = 540,389)	Secondary quantitative data analysis, cross‐sectional study	Biomedical outcomes: transgender men had a significantly higher (>2‐fold) number of cancer diagnoses compared with cisgender men, but not cisgender women. Cancer prevalence among gender nonconforming individuals and transgender women was not significantly different from that of cisgender men and cisgender women. Gender nonconforming survivors had significantly greater physical inactivity, heavy episodic alcohol use, and depression compared with cisgender men and cisgender women. Transgender men and survivors were significantly more likely to report poor physical health and greater medical comorbidities and were less likely to report smoking compared with cisgender men and cisgender women. Transgender women survivors were significantly more likely to report diabetes compared with cisgender men and cisgender women and were more likely to report cardiovascular disease compared with cisgender women	Captures 2, 5–9
Boehmer et al.^19^	USA	*N* = 64, lesbian (*n* = 55), bisexual (*n* = 6), partnered with women (*n* = 3)	Semi‐structured interviews were followed by a self‐administered questionnaire (only survey data were reported in the current study)	Psychosocial outcomes: social support was positively associated with a measure of “fighting spirit” and a reverse association between “fighting spirit” and distress. Disclosure of sexual orientation did not have the anticipated positive outcomes, lack of relationship between disclosure, and lower distress or coping. Women in committed relationships were more likely to disclose to providers. A greater perception of social support was present among women who disclosed to providers. Being in a committed relationship had no positive influence on coping or adjustment. Fatalism and cognitive avoidance were unrelated to distress level. Sexual minority women in this sample experience lower levels of emotional distress compared with the mean mood disturbance score of the scale construction sample (heterosexual population). Time since dx was unrelated to coping and mood measures. Membership in cancer‐support groups was related to higher distress and more cognitive avoidance coping. Care teams should work toward not being heteronormative and understand the burden of coming out. Suggestions included care teams using inclusive screening and assessment for LGBTQ+ adolescent and young adults, educating providers on inclusive language, and creating safe spaces	Captures 5–7, 9
Boehmer et al.^67^	USA	*N* = 122,345, lesbian women (*n* = 918), bisexual women (*n* = 1116) heterosexual women (*n* = 69,078), heterosexual men (*n* = 49,137), gay men (*n* = 1493), bisexual men (*n* = 603)	Cross‐sectional survey	Biomedical outcomes: the prevalence of cancer in LGBTQ+ versus heterosexual population	Captures 2,4–9
Brown and McElroy^20^	USA	*N* = 68; lesbian, bisexual, same‐gender loving (*n* = 54); straight (*n* = 6); queer, questioning, not listed (*n* = 8)	A cross‐sectional quantitative web‐based survey that included open‐ended and closed‐ended response categories	Psychosocial outcomes: SGM breast cancer survivors who chose bilateral mastectomy without reconstruction were generally pleased with their treatment choices and felt it better reflected their gender identity. Considerable inconsistency was reported in provider reactions to this treatment choice, even though participants reported satisfaction with the provider's reactions to disclosing SGM identities	Captures 1–9
Brown and McElroy^21^	USA	*N* = 67, queer identified (*n* = 11), nonqueer identified (*n* = 57)	Mixed methods, cross‐sectional quantitative web‐based survey that included open‐ended and closed‐ended response categories	Psychosocial outcomes: this study did not find any significant differences between queer‐identified and non‐queer‐identified SGM breast cancer survivors in terms of disclosure to providers during cancer diagnosis or treatment. Survey participants reported stress around potential provider reactions to sexual orientation and gender identity (SOGI) disclosure and concerns about provider recognition of their relationships. SGM report feeling uncomfortable discussing their same‐sex relationships in non‐LGBT‐specific groups	Captures 1–9
Desai et al.^22^	USA	*N* = 1025, sexual minority (*n* = 64), heterosexual (*n* = 961)	Quantitative study; cross‐sectional analysis	Psychosocial outcomes: depression: 31% percent of SM participants met the criteria for clinical depression compared with 23% of heterosexual participants (*p* = 0.12); the SM group had significantly higher mean PHQ8 (Patient Health Questionnaire depression) scores compared with the heterosexual group (7.8 vs. 6.0, *p* = 0.009). Anxiety: 34% of SM participants met the criteria for clinical anxiety compared with 20% of heterosexual participants (*p* = 0.004). The SM group had significantly higher mean GAD7 scores compared with the heterosexual group (7.2 vs. 5.7, *p* = 0.039). Social support: SM participants reported significantly less social support compared with heterosexual participants (SM: mean score 35.9; heterosexual: 38.1; *p* = 0.031)	Captures 2,5,6,9
Ebrahim et al.^68^	USA	*N* = 125,691, males (*n* = 99,560), females (*n* = 26,131)	Quantitative; analyzed June 2002 European non‐aggregate acquired immunodeficiency syndrome (AIDS) data set	Biomedical outcomes: cancer as the initial AIDS‐defining illness	Captures 2, 7–9
Estefan et al.^69^	Canada	*N* = 2	Narrative inquiry	Psychosocial outcomes: describes experiences of developing a sense of sexual self while undergoing cancer treatment and survivorship	Captures 2–9
Fish et al.^70^	UK	*N* = 30, lesbians (*n* = 11), gay men (*n* = 15), bisexual men (*n* = 3), queer (*n* = 1)	Qualitative interviews	Psychosocial outcomes: three themes regarding disclosure: authenticity as a driver for disclosure in cancer care, partners as a (potential) salutogenic resource, and creating safe, healing environments conducive to disclosure	Captures 2, 4–9
Gooren et al.^71^	The Netherlands	*N* = 3103, male‐to‐female transsexual patients (*n* = 2307), female‐to‐male transsexual patients (*n* = 795)	Cross‐sectional secondary data set	Biomedical outcomes: breast cancer development following cross‐sex hormone treatment	Captures 2, 7–9
Gorman et al.^23^	USA	*N* = 22, bisexual (*n* = 1), gay/lesbian (*n* = 2), heterosexual (*n* = 19)	Interviewed 11 couples (22 individuals) with a history of breast or gynecologic cancer to review and pretest intervention materials	Biomedical outcomes: reproductive and sexual health concerns but did not report specific outcomes for SGM	Captures 2–5, 7–9
Gurevich et al.^72^	Canada	*N* = 40, Gay (*n* = 1), Heterosexual (*n* = 39)	Semi‐structured interviews	Psychosocial outcomes; Did not report specific outcomes for SGM	Captures 7
Hernandez et al.^73^	USA	*N* = 211 transmasculine Individuals	Tissue sampling	Biomedical outcomes: one patient receiving gender‐affirming top surgery had breast cancer	Captures 2, 5–9
Hutchcraft et al.^24^	USA	*N* = 11,066, heterosexual (*n* = 10,830), lesbian (*n* = 141), bisexual (*n* = 95)	Cross‐sectional survey	Psychosocial and Biomedical outcomes: bisexual women reported higher psychosocial distress than heterosexual survivors	Captures 2, 4–9
Jabson and Bowen^25^	USA	*N* = 211, sexual minority women (*n* = 68), heterosexual women (*n* = 143)	Cross‐sectional online survey	Psychosocial outcomes: SMW (sexual minority women) breast cancer survivors experienced higher levels of stress compared with heterosexual breast cancer survivors	Captures 2, 5–9
Jaffe et al.^26^	USA	*N* = 50 homosexual	Qualitative: interviews	Biomedical outcomes: the number of male sexual partners was associated with Kaposi's sarcoma	Captures 5
Kamen et al.^27^	USA	*N* = 828, LGBT cancer survivors (*n* = 270), matched heterosexual cancer survivors (*n* = 621)	Quantitative: cross‐sectional	Psychosocial outcomes: LGBT cancer survivors reported greater symptoms of depression and greater levels of social/relationship concerns than matched heterosexual cancer survivors. Those identifying as male, gay, bisexual, and transgender cancer survivors reported greater symptoms of depression than heterosexual men cancer survivors	Captures 7
Katz et al.^74^	Canada	*N* = 7, gay men (*n* = 3), lesbian women (*n* = 4)	Interview	Psychosocial outcomes; Body image: alterations to body image caused by cancer treatment. Becoming more conscious about their body, wondering how people are going to perceive the change	Captures 2 and 5
Kerr et al.^75^	Australia	*N* = 12, transmen (*n* = 4), transwomen (*n* = 4), nonbinary (*n* = 3), genderqueer (*n* = 1)	Interview	Psychosocial outcomes: hyperawareness of the body during scans, anxiety, and distress associated with the skin checks coming from uniquely generated bodies and interactions	Captures 2, 5, 7–9
Ketcher et al.^76^	USA	*N* = 12; heterosexual (*n* = 8); bisexual (*n* = 1); lesbian/gay (*n* = 1); queer (*n* = 1), pansexual (*n* = 1)	Secondary analysis of semi‐structured interview data	Psychosocial outcomes: mostly relied on familial support, may lead to a delay or regression in the developmental milestone of YA becoming independent. Peer support was desired, but several barriers to receiving it	Captures 2,3,5–7
Legere and MacDonnell^77^	Canada	*N* = 7, lesbian woman (*n* = 5), bisexual woman (*n* = 1), heterosexual woman (*n* = 1)	Interview	Psychosocial outcomes: heterosexism pervaded interactions with care settings: staff in reception would not put the patient's partner as a partner, but instead put “friend”. The invisibility of resources that explicitly address lesbians and bisexuals further normalize heterosexual relationships and exacerbate barriers to meaningful support from care ex) doctors say they do not need PAP smears, a pamphlet that focuses on resuming heterosexual sexual activity after reproductive cancer post‐surgical care	Captures 2,5,7–9
Letourneau et al.^28^	USA	*N* = 918; Lesbian, bisexual, transgender (*n* = 29); Heterosexual (*n* = 813)	Cross‐sectional survey	Psychosocial and Biomedical outcomes; no sexual minority women preserved their fertility	Captures 2, 5–7
Matthews et al.^78^	USA	*N* = 175, gay/lesbian (*n* = 149), bisexual (*n* = 13), others (*n* = 13)	Cross‐sectional surveys; data were collected via a nationally advertised online short‐form health survey	Psychosocial outcomes: physical and mental HRQoL	Captures 2,5,7–9
McGregor et al.^29^	USA	*N* = 57 lesbian women with breast cancer	Quantitative: cross‐sectional	Psychosocial outcomes: internalized homophobia related to greater distress. The disclosure did not relate to lower distress. Internalized homophobia promotes distress through lower self‐esteem and the perceived unavailability of social support. Low self‐esteem led to internalized homophobia by way of elevated distress. Internalized homophobia related inversely to the utilization of healthcare resources (frequency of pap smears and gynecologic examinations but no association with breast self‐examination)	Capture 4
Parton et al.^79^	Australia	*N* = 693, nonheterosexual (*n* = 13), heterosexual (*n* = 677)	Qualitative: open‐ended questions and semi‐structured interviews	Psychosocial outcomes: three themes were identified: (1) the incomplete woman, (2) the abject, asexual woman, and (3) out of time and social isolation. Did not report specific outcomes for SGM	Capture 1
Paul et al.^80^	USA	*N* = 13 lesbian and/or bisexual women	Qualitative semi‐structured interviews	Psychosocial outcomes: heteronormativity that is implicit in the structure of support resources can serve as a barrier to support for SMWs and their partners. Participants emphasized the value of cancer support groups and resources tailored to SMW while stating that other dimensions of identity or experience, particularly age and cancer stage, were also important. Participants noted the dearth of social support resources for same‐sex partners. Family of origin and partners were typically participants' primary sources of tangible and emotional support; participants often engaged in protective buffering to mitigate caregivers' distress. Single women faced the greatest challenges in terms of support needs and resources. Former partners were often key sources of support Flexibility in relationship roles enabling some SMWs to include former partners as significant means of support may be a source of resiliency, particularly for unpartnered SMW cancer patients	Captures 2,5‐7,9
Rosser et al.^81^	USA	*N* = 205; lesbian, gay, or homosexual (*n* = 5); straight or heterosexual (*n* = 179); bisexual (*n* = 5); something else (*n* = 5); do not know (*n* = 2); choose not to disclose (*n* = 7); nonresponse (*n* = 2)	Cross‐sectional	Psychosocial outcomes: acceptability and feasibility of SOGI questions	Captures 2, 5,7–9
Rubin and Tanenbaum^82^	USA	*N* = 13 lesbian and/or bisexual women	Interview	Psychosocial outcomes: five themes were identified: reconstruction decision‐making, gender policing and medicalization, negotiating the politics of breast reconstruction, deconstructing and reconstructing womanhood, breast reconstruction, and terror management	Captures 1, 2
Russell et al.^30^	USA	*N* = 56, LGBTQ (*n* = 22), heterosexual (*n* = 34)	Semi‐structured telephone interviews	Psychosocial outcomes: cancer treatments' effects on relationships, plans for parenthood, and fertility preservation decision‐making	Captures 2, 3, 5, 7–9
Sinding et al.^83^	Canada	*N* = 26, lesbian (*n* = 22), gay (*n* = 2), dyke (*n* = 1), bisexual (*n* = 1)	Participatory action research (PAR)	Psychosocial outcomes: heterosexism in cancer care: uncomfortable interactions with the healthcare professionals. Aspects of lesbian identity and social contexts directly relevant to cancer care were sometimes dismissed or overlooked by health professionals. Lack of lesbian‐positive psychological support and denial of standard care	Captures 2, 7
Sinding et al.^31^	Canada	*N* = 26, lesbian (*n* = 22), gay (*n* = 2), dyke (*n* = 1), bisexual (*n* = 1)	Interview	Psychosocial outcomes: most participants experienced robust and competent community support, participants also reported instances of isolation and disconnection linked to fear of cancer, homophobia in the broader community, and patterns of exclusion within lesbian communities	Captures 1, 2, 4, 6, 7
Taylor and Bryson^84^	Canada	*N* = 10; female‐to‐male (*n* = 2); transman (*n* = 3); genderqueer (*n* = 1); gender fluid (*n* = 1); butch (*n* = 1); butch and gender nonconforming (*n* = 1); genderqueer, trans, 2‐sprit, and butch (*n* = 1)	Interview	Psychosocial outcomes: a sense of gender closely tied to experiences with cancer treatment	Captures 1–9
Wide et al.^85^	Sweden	*N* = 989 homosexual (*n* = 12), heterosexual (*n* = 930), bisexual (*n* = 41), other (*n* = 6)	Cross‐sectional	Psychosocial outcomes: among women, identifying as heterosexual and being childless at diagnosis increased the likelihood of recalling receipt of fertility preservation information	Captures 5, 9

Only one study reported on other biomedical outcomes for SGM AYAs.^23^ Other studies capturing biomedical outcomes reported on a broader age range of SGM cancer survivors and addressed general physical health, fertility, cardiovascular disease, and AIDS‐defining cancers that a person with human immunodeficiency virus (HIV) is at high risk of developing. SGM survivors reported significantly greater physical inactivity, compared with cisgender women and men.^16^ SGM survivors, especially transgender men survivors, were significantly more likely to report poor physical health and greater medical comorbidities; and transgender women survivors were significantly more likely to report cardiovascular disease compared with cisgender counterparts.^18^ Although there was no significant association between sexual orientation and referral to counseling for reproductive compromise after treatment, no women identifying with a known sexual orientation other than heterosexual preserved their fertility.^28^ Last, lesbians reported greater tobacco use and bisexual women reported lower odds of having recent mammography.^24^

### 3.3 Psychosocial outcomes

No psychosocial intervention studies were found (Table 3). When investigating psychosocial outcomes, some studies reported the outcomes of SGM AYA cancer survivors compared to their heterosexual counterparts. Thirty‐one percent of sexual minority participants met the criteria for clinical depression and 34% met the criteria for clinical anxiety, while 23% of heterosexual participants met the criteria for depression and 20% for anxiety.^22^ It follows that the sexual minority group had a significantly higher mean of depression and anxiety scores compared with the heterosexual group.^22^ Likewise, bisexual women reported higher psychosocial distress than heterosexual women survivors.^24^ Sexual minority women who are breast cancer survivors experienced higher levels of stress compared to heterosexual breast cancer survivors.^25^ Men who identify as gay, bisexual, and transgender cancer survivors reported greater symptoms of depression than cis‐heterosexual male cancer survivors.^27^ Conversely, sexual minority women in the study sample experienced lower levels of emotional distress compared with the mean mood disturbance score of the scale construction sample which was the heterosexual population.^19^

Regarding social support, many studies demonstrated unmet needs or inadequate social support among SGM cancer survivors. SGM participants reported unmet needs for cancer‐related social support for themselves and for their partners, although few reported disclosing their sexual orientation or gender identity to healthcare providers.^21^ LGBT cancer survivors reported greater symptoms of depression and greater levels of social/relationship concerns than matched heterosexual cancer survivors.^26^ Internalized homophobia among lesbian women treated for early stage breast cancer was associated with greater distress through low self‐esteem and perceived unavailability of social support.^29^ Moreover, one study found that SGM patients with worse access to care have poorer quality of life (QOL) than non‐SGM cancer survivors,^17^ and another study reported that instances of isolation and disconnection reinforced fear of cancer and patterns of exclusion among lesbians.^31^

### 3.4 Quality of the evidence

Results from employing JBI critical appraisal tools revealed that all studies in our final review were of high quality, meeting every criterion for trustworthy and relevant findings for their respective research design. Piloting our conceptual framework of foundational progress in SGM AYA research, each co‐author calculated frequency ratings of their assigned publications according to the presence or absence of aspects within nine domains (Figure 1). Results show that the most frequently addressed domains were *Implications* (*n* = 29), *Nonbinary* (*n* = 28), and *Affirming terminology* (*n* = 27).

## 4 DISCUSSION

Our findings in the current scoping review reveal a limited, but growing empirical literature that provides some evidence of disparities in biomedical outcomes and psychosocial outcomes among SGM AYAs. The resulting body of literature was characterized by large gaps in knowledge on cancer outcomes and on outcomes for distinct sub‐groups within the SGM AYA patient population.

### 4.1 Biomedical outcomes

SGM participants reported greater physical inactivity, greater medical comorbidities, greater participation in high‐risk behaviors such as alcohol use, and poorer physical health compared with non‐SGM controls.^18^ This finding may be attributed to conditions associated with SGM status that either predate or may occur irrespective of a cancer diagnosis. For example, SGM in the general population report negative medical experiences, perceive themselves as discriminated against by providers, and have historically low rates of healthcare use.^32^, ^33^ As such, SGM AYAs' outcomes may be disadvantaged by a lack of adequate comprehensive health care prior to their cancer diagnosis. It follows that SGM AYAs may have greater potential for treatment and survivorship nonadherence due to prior negative healthcare experiences that predate their diagnosis of cancer. It is concerning that SGM AYA survivors utilize cancer prevention services, such as mammography with less frequency than their non‐SGM peers as a presentation with the later‐stage disease in childhood cancer survivors is associated with poor outcomes.^25^, ^34^

The finding that bisexual women reported lower odds of recent mammography aligns with the empirical literature that identifies patterns of delayed or declined cancer screening among marginalized groups, especially among those at the intersection of multiple minoritized statuses (e.g., SGM and racial/ethnic minorities).^35^, ^36^, ^37^, ^38^ Future studies are advised to test interventions that mitigate stigma and fear of discrimination—two known barriers to receiving prophylactic screenings among these groups.

### 4.2 Psychosocial outcomes

The finding that SGM AYAs experienced greater clinical depression, anxiety, and psychosocial distress than cis‐heterosexual AYAs reflects literature that substantiates minority stress theory among marginalized populations.^39^, ^40^, ^41^, ^42^, ^43^ While there are higher rates of psychosocial distress in cancer survivors compared with the general population, there are also higher rates of psychosocial distress in the SGM population compared with the general population.^44^, ^45^ The extent to which a cancer diagnosis further exacerbates psychosocial distress in SGM AYA survivors compared with SGM individuals who have not had cancer needs to be assessed.

Further research is needed to develop necessary interventions to alleviate the lifelong impacts of stress processes (e.g., experience of prejudice, expectations of rejection, internalized homophobia) on SGM AYA psychosocial outcomes during and after treatment. A social genomics approach to minority stress among SGM AYAs is needed to further understand the relationship between social determinants of health and cancer's biologic mechanisms.^46^ The social determinants of health are defined as the economic, education, healthcare access, built environment, and social conditions in which people live and have been identified as widely contributing to health disparities and inequities.^47^

The finding that SGM survivors reported a lack of social support, experiences of isolation or disconnection, and poorer QOL is consistent with literature that demonstrates a lack of appropriate formalized support for SGM AYAs.^8^, ^27^, ^48^, ^49^, ^50^, ^51^, ^52^ Future research is advised to tackle gaps in patient‐provider communication and peer‐to‐peer support programming such that service delivery is appropriately and equitably tailored to LGBTQ+ identity concerns.^53^ Furthermore, it is important to understand and incorporate modes of social support among queer communities that exist beyond formalized medical and psychosocial services. A deep tradition of community care and mutual aid—stemming from medical neglect during the HIV/AIDS epidemic—is often upheld among LGBTQ+ groups who divest from formal care due to negative experiences.^54^, ^55^

### 4.3 Gaps in research

**FIGURE 3:**
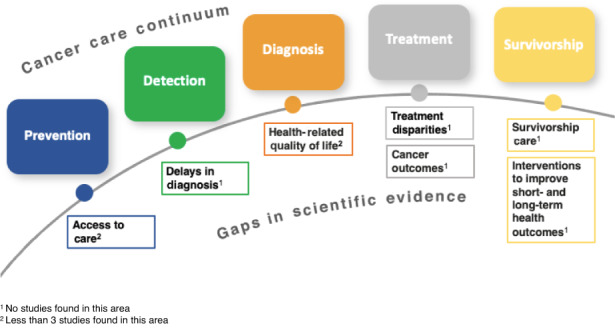
Gaps in scientific evidence of sexual and gender minority Adolescent and young adults along the cancer care continuum. Adapted from National Institutes of Health.^56^

Although survival outcomes are the most common primary endpoints in cancer clinical trials, none of the included studies addressed survival outcomes. No studies assessed overall or event‐free survival for SGM AYAs for any prevalent cancer type seen in the AYA population. Although recent studies have highlighted racial, ethnic, and socioeconomic disparities in survival for AYAs diagnosed with cancer, the fact that survival outcomes have not been assessed in SGM AYAs is highly concerning. Figure 3 illustrates gaps in scientific evidence on major aspects of SGM AYAs' care and outcomes along the National Cancer Institute's cancer care continuum.

Most studies examined AYAs as part of a larger study sample with a broader age range (86.5%, *n* = 32) and much fewer studies (13.5%, *n* = 5) focused exclusively on AYAs. Articles that were published in 2020 or later were more likely to include dimensions of gender—that is, nonbinary, transgender—in addition to sex and sexual orientation demographic items. Despite the acknowledgment of noncisgender participants, a critical gap exists in focusing on transgender, genderqueer, gender diverse, and intersex PRO.^57^ Only 16 (43.2%) studies included participants who identify as such.

The findings of these studies demonstrate disparate biomedical and psychosocial health outcomes of SGM AYAs compared with their cis‐heterosexual counterparts when these outcomes were assessed and reported. Unfortunately, most often studies did not collect or report data on sexual orientation and/or gender identity, and when studies did collect this data, results for AYAs were often combined with data from older SGM survivors.

While these results are critical to address, equal attention to aspects of queer resilience is owed. In some cases, SGM AYAs are less negatively impacted by the psychosocial effects of cancer and treatment. For example, breast cancer survivors who embrace the choice to “go flat”—that is, opt out of reconstruction after bilateral mastectomy—were more likely to be genderqueer than cisgender, and these patients found comfort and pride in having modified breasts that more closely aligned with their gender identities.^20^ Also, SGM AYAs experienced less romantic partnership distress about infertility arising from cancer than their cis‐heterosexual counterparts.^30^ Elevating resiliency through a strengths‐based lens is of tantamount importance to identifying and eradicating disparate biomedical and psychosocial outcomes for SGM AYAs.

### 4.4 Conceptual framework of foundational progress in SGM AYA research

We developed and piloted a conceptual framework to assess the quality of scientific evidence on SGM AYAs that derive from Levin et al.'s framework to assess sex, sexual orientation, and gender orientation in oncology research.^14^ As shown in Figure 1, our framework reflects Levin et al.'s tripart schema that considers defining the population, measuring the population, and translating research to clinical services, while elaborating upon nine essential domains for high‐quality research studies that capture SGM AYAs.

Specifically, *Accurate terminology* indicates that the study utilizes accurate sexual orientation/gender identity (SO/GI) terminology for today's cohort of SGM AYAs. *Dimensions of human sexuality* signify that the study correctly utilizes the three dimensions of human sexuality as it relates to the study. *Nonbinary* means that the study utilizes more than two identities to identify SOGI. *Goal of study* suggests that the study focuses its primary endpoint on SGM AYA cancer patients. *Affirming terminology* indicates that the study utilizes affirming SGM terminology that applies to the current cohort of AYAs.

Furthermore, *Stakeholder collaboration* denotes that the study discloses collaboration on research activities with SGM AYA patients and/or SGM AYA stakeholders in either the research methods or authors' affiliation. *Multiple methods to collect SGM identity* confirm that the study captures SGM identity via methods that offer both close‐ended and open‐ended response options. *Implications* indicate that the study offers practice and/or policy implications that directly address SGM AYA health. Last, *Validated frameworks* verify that the study utilizes instruments that are validated for SGM AYAs.

Findings revealing the most frequently included domains of SGM AYA research point to trends in research to date in the areas of *Implications*, *Nonbinary*, *and Affirming terminology*. These frequencies suggest that studies in the sampling trend toward providing practice and/or policy implications based on the empirical findings, which bodes well for efficiently translating findings into improved service delivery. A high prevalence of utilizing *Nonbinary* criteria indicates that studies in the sample measure SOGI as spectrum‐based constructs, and that authors produce demographic data accordingly by soliciting more than one, nondichotomous response category for each identity domain. Similarly, a notable presence of *Affirming terminology* suggests a cultural shift toward endorsing identity affirmation^58^ among SGM respondents by employing scientific language that adequately reflects the population of interest.

The least observed domains included *Stakeholder collaboration* (*n* = 11), *Multiple methods to collect SGM identity* (*n* = 9), and *Validated measures* (*n* = 6). Increased stakeholder collaboration between academic investigators and community‐based stakeholders is needed. Methodologically, increasing the practice of collecting demographics using multiple methods (e.g., closed‐ and open‐ended response modalities) would improve the production of rigorous and nuanced descriptive data^14^, ^59^, ^60^. Additionally, establishing validated instruments, questionnaires, theories, and frameworks that address the needs and experiences of individuals living at the intersection of SGM and AYA and their identities is critical to advancing the field. To the best of our knowledge, no validated survey instruments to assess SGM AYAs' experiences currently exist.

### 4.5 Strengths and limitations

This scoping review did not include studies that focused on healthcare providers. Future systematic reviews are encouraged to examine literature that addresses the needs and experiences of our SGM AYA healthcare providers and other stakeholders.

Our study harkens a paradigmatic shift toward inclusive, innovative, and interdisciplinary cancer research with SGM.^61^ This interdisciplinary author team comprises researchers with expertise in AYA oncology, social work, information science, and anthropology. Furthermore, the author team includes “embodied researchers”^62^, ^63^ whose lived experiences as patient‐scientists, physician‐scientists, and/or SGM individuals reflect the topics of study. Future author teams are encouraged to incorporate embodied researchers whose diversity of lived experiences reflects upon the literature in question in purposeful and ethical ways.^64^

## 5 CONCLUSION

The current study is the first to systematically identify, synthesize, and critique the literature to date on SGM AYAs by developing and piloting a conceptual framework for foundational progress to critically appraise the quality of each contribution. Results point to gaps in scientific evidence on SGM AYAs at numerous points across the cancer care continuum. Future efforts should fill this void with high‐quality empirical studies that reveal unknown disparities in care and outcomes and are inclusive of the intersectionality of SGM AYAs with other minoritized experiences, thereby advancing health equity in meaningful ways.

## AUTHOR CONTRIBUTIONS

**Christabel K. Cheung:** Conceptualization (lead); data curation (lead); formal analysis (lead); investigation (lead); methodology (lead); project administration (lead); writing – original draft (lead); writing – review and editing (lead). **Haelim Lee:** Conceptualization (supporting); data curation (supporting); formal analysis (supporting); funding acquisition (supporting); investigation (supporting); methodology (supporting); project administration (supporting); writing – original draft (lead); writing – review and editing (supporting). **Nina Jackson Levin:** Data curation (equal); formal analysis (equal); writing – original draft (lead); writing – review and editing (supporting). **Eunju Choi:** Data curation (equal); formal analysis (equal); writing – original draft (lead); writing – review and editing (supporting). **Valentina A. Ross:** Data curation (equal); formal analysis (equal); writing – review and editing (supporting). **Yimin Geng:** Data curation (lead). **Bria N. Thomas:** Visualization (lead); writing – review and editing (supporting). **Michael E. Roth:** Conceptualization (lead); data curation (supporting); formal analysis (supporting); investigation (supporting); writing – original draft (supporting); writing – review and editing (lead).

## FUNDING INFORMATION

M. Roth received research support from the National Cancer Institute (P30 CA016672). N. Levin received research support from the National Cancer Institute institutional training (grant T32‐CA‐236621). The content is solely the responsibility of the authors and does not represent the official views of the National Institutes of Health or the National Cancer Institute. N. Jackson Levin also received research support from the University of Michigan Vivian A. and James L. Curtis School of Social Work Center for Health Equity Research and Training, Signature Programs Initiatives. Additionally, the University of Maryland School of Social Work's Doctoral Research Assistant program provided research support for H. Lee and its Research Assistant Scholars Program provided research support for V. Ross.

## CONFLICT OF INTEREST STATEMENT

The authors have no conflict of interest.

## Data Availability

Data sharing is not applicable to this article as no new data were created or analyzed in this study.
